# Developing a simulation-based education curriculum sample in postgraduate emergency medicine education

**DOI:** 10.3389/fmed.2026.1755603

**Published:** 2026-02-19

**Authors:** Mevlüt Okan Aydin, Sinem Yıldız, Cem Oktay, Maruf Beğenen, Sevilay Ayas, Hatice Gülbas

**Affiliations:** 1 Department of Medical Education, Faculty of Medicine, Bursa Uludag University, Bursa, Türkiye; 2 Department of Medical Education, Faculty of Medicine, Marmara University, Istanbul, Türkiye; 3 Department of Emergency Medicine, Faculty of Medicine, Akdeniz University, Antalya, Türkiye; 4 Gemlik State Hospital, Bursa, Türkiye; 5 Bursa Provincial Health Directorate, Bursa, Türkiye

**Keywords:** clinical skills, emergency medicine, medical education, residency training, simulation

## Abstract

**Objectives:**

This study aims to develop and conduct a preliminary evaluation of a simulation-based curriculum in emergency medicine residency training. Simulation provides medical students with the opportunity to experience real-life scenarios in a controlled environment, enhancing skills such as critical thinking, problem-solving, and decision-making.

**Methods:**

The research was conducted at Bursa Uludağ University, Faculty of Medicine, and consists of a mixed-methods study in three phases. In the first phase, data were collected in three rounds from emergency medicine specialists in Türkiye using the Delphi Technique. In the second phase, a multidisciplinary workshop was held to develop the content of a sample simulation-based learning program using the collected data. In the third phase, the developed simulation scenario was implemented using the multi-patient technique. Four months post-implementation, qualitative data were collected through focus group meetings involving 13 of these residents. The data from the focus groups underwent thematic analysis.

**Results:**

Analysis of the qualitative data indicated that participants perceived improvements in their practical skills and clinical decision-making abilities. Performance evaluations were supported by focus group discussions conducted after the simulation exercises. Post-simulation focus group discussions indicated that participants found value in the training and reported benefits in transferring their simulation experiences to real-life situations.

**Conclusion:**

This study suggests that simulation-based learning can be a valuable addition to emergency medicine residency education. This educational method has the potential to enhance training quality by providing residents with opportunities for safe practice, immediate debriefing, and performance reflection, which may contribute to their confidence and competence.

## 1 Introduction

Simulation-based learning (SBL) has emerged as a transformative approach in medical education, recreating real-life scenarios to provide a controlled, risk-free environment for developing clinical skills, critical thinking, and decision-making competencies (1). With roots in aviation and military training, SBL has become a cornerstone of modern healthcare education, effectively bridging the gap between theoretical knowledge and clinical practice. This value is further underscored during the COVID-19 pandemic, when traditional clinical training was disrupted (2). Its adoption spans diverse disciplines, from anesthesiology to emergency medicine, driven by evolving clinical practices, time constraints in training, and heightened medicolegal awareness (3, 4). Simulations vary in complexity and fidelity, ranging from low-tech task trainers for intravenous catheterization to high-fidelity manikins capable of mimicking physiological responses, such as breathing, pupillary reflexes, and drug interactions (5). These tools are deployed in simulation centers, *in situ* settings, or remotely, tailored to meet specific learning objectives (6, 7). Studies consistently highlight SBL’s efficacy in enhancing technical proficiency, reducing medical errors, and improving patient outcomes, while its cost-effectiveness encourages the implementation of structured, phased programs that optimize resource utilization (8).

Postgraduate medical education, particularly in high-stakes fields like emergency medicine, has increasingly integrated SBL to address the limitations of traditional apprenticeship models. For emergency medicine, where rapid decision-making is paramount, trauma-focused simulations have proven effective in closing experience gaps, particularly in resource-limited settings (9). Residency programs demand not only procedural expertise but also competencies in crisis management, interdisciplinary communication, and leadership, skills ideally cultivated through repetitive, feedback-driven simulations (10). Illustrative of this approach, Beirut University’s emergency medicine curriculum implements a structured 12-h simulation program based on Kern’s model, which is tailored to teach foundational skills to junior residents and advanced resuscitation and team management to senior trainees (11). However, challenges persist, including insufficient instructor training, financial constraints, and technological barriers, as evidenced in Canadian programs where these factors hinder broader implementation (12).

Curriculum development for SBL requires systematic frameworks to ensure alignment with educational goals. Kern’s six-step model, encompassing needs assessment, objective setting, content design, stakeholder engagement, implementation, and evaluation, provides a robust structure for creating context-specific, learner-centered programs (13). Its iterative nature allows for continuous refinement based on multimodal feedback, including self-assessment, observational data, and neurophysiological metrics (14). This study aims to design and evaluate a simulation-based curriculum for emergency medicine residents, utilizing Kern’s model to address identified gaps in procedural and non-technical skill training. By synthesizing evidence on SBL’s pedagogical strengths, cost considerations, and adaptability, the program seeks to enhance clinical readiness while navigating implementation challenges. The findings will contribute to the growing body of research on optimized simulation training frameworks, ultimately supporting safer, more competent healthcare delivery.

## 2 Materials and method

### 2.1 Study design

This study employed an exploratory, sequential, mixed-methods design across three phases, with the aim of developing and evaluating a simulation-based learning curriculum for postgraduate emergency medicine (EM) education. The study was approved by the Bursa Uludağ University Clinical Research Ethics Committee (Approval No: 2019-19/27, Date: 20.11.2019), aimed to develop a simulation-based learning program for emergency medicine (EM) residents. Participation was voluntary and required written consent. Anonymity and confidentiality were maintained throughout data collection and analysis.

In Phase 1, a quantitative needs analysis was conducted using the Delphi technique to identify core competencies and priority content areas for simulation-based training. Phase 2 involved program design through a collaborative expert workshop, informed by the Delphi findings. Phase 3 consisted of curriculum implementation using integrated simulation scenarios, followed by focus group discussions to explore participants’ experiences, perceived learning transfer, and contextual factors influencing feasibility and acceptability.

Although objective performance data were collected during the implementation phase, detailed quantitative outcomes were reported separately in a prior publication focusing on simulation effectiveness (15). Accordingly, the present manuscript emphasizes the qualitative component, using thematic analysis to expand upon and contextualize the curriculum development process and participants’ reflections on the simulation experience. Integration of quantitative and qualitative strands occurred at the interpretation level, whereby qualitative findings were used to explain how and why the simulation curriculum addressed the training needs identified during the initial quantitative phase.

#### 2.1.1 Phase 1: needs analysis via Delphi technique

A three-round Delphi survey was administered to 56 EM specialists from clinics across Turkey to identify simulation-based learning priorities. Participants rated 93 EM competency topics [derived from the European Society for Emergency Medicine Framework V.2 (16)] using a 5-point Likert scale (1: No need for simulation to 5: Essential need for simulation). The categories included Diagnoses and Syndromes, Procedural Skills/Diagnostic Tests, and Professional Attitudes and Behaviors.

The Delphi technique, a structured method for achieving expert consensus through iterative feedback (17), was applied. In Round 1, items scoring <4 were eliminated. Remaining items were re-evaluated in Rounds 2 and 3 until consensus (≥4) was reached (Table 1). Finalized topics (e.g., Pulmonary Emergencies, Trauma Emergencies) were used in Phase 2 (Table 2). This approach aligns with prior Delphi studies on competency prioritization in healthcare education (18).

**Table 1:** Changes made to the training content during the Delphi method rounds.

Topic category	Delphi Round Number of participants (EM specialists)								
	1. Round *n* = 56			2. Round *n* = 36			3. Round *n* = 28		
	Topics at start *n* (%)	Topics eliminated *n* (%)	Topics retained *n* (%)	Topics at start *n* (%)	Topics eliminated *n* (%)	Topics retained *n* (%)	Topics at start *n* (%)	Topics eliminated *n* (%)	Topics retained *n* (%)
Diagnosis and syndromes	*n* = 18 (100%)	*n* = 12 (66.6%)	*n* = 6 (33.3%)	*n* = 6 (33.3%)	*n* = 1 (5.5%)	*n* = 5 (27.7%)	*n* = 5 (27.7%)	*n* = 0 (0%)	***n* = 5** **(27.7%)**
Procedural skills/ diagnostic tests	*n* = 16 (100%)	*n* = 1 (6.6%)	*n* = 15 (93.4%)	*n* = 15 (93.4%)	*n* = 5 (31.2%)	*n* = 10 (62.5%)	*n* = 10 (62.5%)	*n* = 4 (25%)	***n* = 6** **(37.5%)**
Professional attitudes and behaviors	*n* = 3 (100%)	*n* = 0 (0%)	*n* = 3 (100%)	*n* = 3 (100%)	*n* = 0 (0%)	*n* = 3 (100%)	*n* = 3 (100%)	*n* = 0 (0%)	***n* = 3** **(100%)**

Bold values indicate the number of topics remaining in each category at the end of the 3rd round.

**Table 2:** Evolution of curriculum topics through the Delphi process: Initial comprehensive list and final selected topics for the simulation-based learning program following the third round.

Initial content	Final selection
Diagnosis and syndromes	Diagnosis and syndromes
Cardiovascular emergencies	Cardiovascular emergencies
Dermatologic emergencies	Circulatory and vascular emergencies
Endocrine, metabolic, and autoimmune emergencies	Gynecological and obstetric emergencies
Circulatory and vascular emergencies	Pulmonary emergencies
Ear, Nose, and throat (ENT) emergencies	Trauma
Oral and neck emergencies	Procedural skills/diagnostic tests
Gastrointestinal/hepatobiliary/pancreatic emergencies	Cardiopulmonary resuscitation (CPR)
Gynecological and obstetric emergencies	Emergency airway management
Hematological and oncological emergencies	Assessment of breathing and ventilation
Infectious disease emergencies	Circulation management
Musculoskeletal emergencies	Vascular access procedures
Neurological emergencies	Obstetric/gynecological procedures
Ophthalmic emergencies	Critical patient transport skills
Pulmonary emergencies	Wound care
Renal and urological emergencies	Emergency imaging modalities
Trauma	Disaster medicine
Environmental emergencies	Professional attitudes and behaviors
Toxicology	Leadership (crisis resource management, team leadership)
Psychiatric emergencies	Team membership (interdisciplinary collaboration)
Procedural skills/diagnostic tests	Effective communication skills
Cardiopulmonary resuscitation (CPR)	
Emergency airway management	
Assessment of breathing and ventilation	
Circulation management	
Vascular access procedures	
ENT procedures	
Gastrointestinal procedures	
Genitourinary procedures	
Musculoskeletal procedures	
Neurological procedures and skills	
Obstetric/gynecological procedures	
Ophthalmic procedures and skills	
Critical patient transport skills	
Wound care	
Emergency imaging modalities	
Disaster medicine	
Professional attitudes and behaviors	
Leadership (crisis resource management, team leadership)	
Team Membership (interdisciplinary collaboration)	
Effective communication skills	

#### 2.1.2 Phase 2: program design workshop

A three-day workshop was organized at Bursa Uludağ University, involving 15 EM specialists (including two international experts from the U.S.) and five medical education specialists. Participants were divided into five groups, each comprising EM faculty, medical educators, residents, and medical students. Groups used ACGME (Accreditation Council for Graduate Medical Education) EM competency frameworks to design 2 simulation scenarios for each assigned topic (e.g., Cardiac Emergencies, Obstetric/Gynecologic Emergencies).

Guided by standardized templates, groups defined learning outcomes, scenario flow, and assessment criteria. On Day 3, draft scenarios were refined via consensus discussions. Finalized scenarios were reviewed by the research team and converted into integrated multi-patient simulations for Phase 3.

#### 2.1.3 Phase 3: integrated simulation and focus group evaluations

Five simulation scenarios were piloted with 15 EM residents (post-Year 1 trainees). Each participant completed a 21.2-min session, which was evaluated by two independent EM specialists using checklists (1–5 scale) aligned with the learning outcomes. Post-simulation, residents participated in structured reflection/feedback sessions and transcribed them for qualitative analysis.

Four months post-simulation, four focus groups (3–5 participants each) explored residents’ experiences. Sessions, conducted online and moderated by two researchers, lasted 75 min. Audio-visual recordings were transcribed and thematically analyzed to identify patterns in perceptions of simulation utility and integration into clinical practice.

### 2.2 Study population and sampling

Phase 1: All EM residency programs in Turkey (*N* = 123 clinics) were eligible; 56 specialists participated.

Phase 2: Convenience sampling recruited 15 EM specialists and five educators with simulation expertise.

Phase 3: All EM residents at Bursa Uludağ University (*N* = 15) were included, excluding first-year trainees.

### 2.3 Data analysis

Quantitative data in this study were derived from responses to the Delphi method survey. Qualitative data consisted of transcriptions from post-simulation individual reflection/feedback sessions and focus group interviews. Quantitative analyses were conducted using Jamovi 2.3.21 software to calculate frequencies, percentages, means, and standard deviations.

Focus groups, a qualitative research method, involve moderated discussions among small groups to explore collective experiences and perspectives on specific topics (19). This approach facilitates intensive participant interaction, enabling researchers to uncover shared viewpoints, perceptions, and insights. This method is particularly effective for investigating socially contextualized issues, evaluating attitudes, identifying trends, and assessing programs or services (20).

Thematic analysis, a qualitative research framework, was applied to identify and interpret recurring themes or patterns within the data (21). This process involved systematic coding to derive common concepts, ideas, or themes emerging from participants’ narratives, allowing for nuanced interpretation of collective experiences (21).

To ensure rigor and transparency in the analysis process, the following steps and measures were implemented:

Familiarization: Two researchers (M. O. A. and S. Y.) independently read and re-read all transcripts to immerse themselves in the data.
Initial coding: The same two researchers independently generated initial codes for the first three transcripts. They then met to compare their coding frameworks, discuss discrepancies, and develop a preliminary consensus codebook.
Systematic coding: Using the refined codebook, one researcher (S. Y.) coded the entire dataset. The second researcher (M. O. A.) independently coded a 30% sample of the transcripts. Coding disagreements were resolved through discussion and consensus during regular analytical meetings, with reference back to the original transcripts. This process enhanced the consistency and credibility of the coding.
Theme generation and review: The researchers collaboratively sorted codes into potential themes, reviewing and refining them iteratively against the coded data and the entire dataset to ensure they accurately represented the participants’ experiences.
Defining and naming themes: Final themes were clearly defined and named to capture their essence.
Data saturation: Data saturation was deemed achieved when the analysis of subsequent transcripts yielded no new substantive codes or themes, and the existing themes were richly developed and recurrent.
Reflexivity and audit trail: The research team maintained reflexivity by acknowledging their positions as clinical educators and discussing potential biases during analysis meetings. An audit trail was kept, including raw transcripts, coded data, thematic maps, and analytical meeting notes, to ensure the decision-making process was transparent and traceable.

## 3 Results

### 3.1 Phase 1: needs analysis

A total of 56 emergency medicine (EM) specialists from 31 clinics participated in the first phase (54% male, *n* = 30; 46% female, *n* = 26). The mean age was 41.7 years (SD = 2.1), with an average teaching experience of 8.4 years (SD = 0.45). Participants’ clinics reported an average of 7.4 educators (SD = 1.1) and 27.2 EM residents (SD = 1.3), resulting in a faculty-to-resident ratio of 3.67 (SD = 0.34).

Among participants, 73.8% (*n* = 41) reported prior experience delivering simulation-based learning. The most frequently used method was low-fidelity simulations (76.4%, *n* = 42), while simulations for complex procedural learning were the least common (27.3%, *n* = 15).

Through the Delphi method, consensus was achieved over three rounds (Table 1). The first round commenced with 56 specialists. Response rates for the subsequent rounds were 64.3% (*n* = 36) for the second round and 50% (*n* = 28) for the third and final round. Initial content elimination rates were 72.3% for Diagnoses and Syndromes, 62.5% for Procedural Skills/Diagnostic Tests, and none for Professional Attitudes and Behaviors. Final prioritized topics (e.g., Pulmonary Emergencies, Trauma Emergencies) were retained for Phase 2. The initial comprehensive list and the final topics are detailed in Table 2.

### 3.2 Phase 2: program development workshop

Fifteen EM specialists and five medical educators developed simulation scenarios using standardized templates. Groups designed two scenarios per assigned topic (e.g., Cardiac Emergencies, Vascular Emergencies), aligning learning outcomes with ACGME competencies. Draft scenarios were refined through consensus discussions and finalized by the research team, forming the basis for Phase 3 simulations.

### 3.3 Phase 3: simulation implementation and evaluation findings

#### 3.3.1 Simulation performance

In this phase, 15 emergency medicine residents (7 female, 8 male) with 18–45 months of specialization training experience at Bursa Uludağ University Faculty of Medicine Department of Emergency Medicine participated in simulation applications and semi-structured debriefing sessions using finalized scenarios developed during the first two stages of the study. Objective performance measures were collected during simulation implementation and are reported separately in a prior publication focusing on simulation effectiveness (15).

#### 3.3.2 Thematic analysis of post-simulation focus group interviews

The perceptions of emergency medicine residents (*n* = 13) regarding their simulation-based learning experience were collected through focus group interviews conducted 4 months after the training and analyzed using thematic analysis. The analysis identified six main themes and eleven sub-themes.

##### 3.3.2.1 Focus on experience

This theme, reflecting the core dynamics of the emergency department, consisted of three sub-themes:

Teamwork and leadership: Participants described the perceived critical importance of leadership in managing clinical scenarios, noting its potential to mitigate medical errors, particularly when working with inexperienced teams. One participant stated, “…in every department, the physician is the ultimate psychological authority in the emergency department as well. That physician must assume all responsibility there” [F. C.], while another expressed, “If you are working with a team you have never seen or met before… you need to be a bit more careful” [D. D.].
Workload and multitasking: Participants reflected on the challenges of divided attention in high-intensity environments, suggesting it could detract from a patient-centered approach. One participant summarized this situation by stating, “…we often don’t have much time to think. Because we do some things subcortically” [T. A.].
Communication with patients/relatives: The analysis highlighted participant accounts of conflict between the pressure from patients’ relatives and medical principles. One participant remarked, “In the emergency department… we sometimes find ourselves proceeding with unnecessary tests due to the pressure and manipulation from patients’ relatives” [D.Ö.], while another expressed it as, “This conflict is draining… trying to do what you believe is right for the patient’s well-being, while simultaneously having to consider other factors, is exhausting” [Ö. Y.].

##### 3.3.2.2 Emotions

Participants described a powerful affective impact from the simulation, which they associated with enhanced retention of learning.

Negative emotions: Feelings of fear and guilt associated with making mistakes were described by participants as contributing to motivation. One participant stated, “You feel a realistic fear and guilt… This ensures the knowledge is much more memorable” [F. C.].

Positive emotions: The opportunity for self-critique in a safe environment was reported to reinforce learning and was linked by participants to improved self-confidence. One participant shared the view that, “you do a self-critique, and when you do your own self-critique, you learn better” [D. D.], while another stated, “I used to feel inadequate… afterwards… I gained self-confidence” [T. Z.].

##### 3.3.2.3 Transfer of learning to real practice

Participants frequently reported applying insights from the simulation to their clinical practice.

Transfer: Several participants emphasized that they could recall and apply the simulated experience during real cases. Referring to a real case where he dissuaded a patient’s relative from an unnecessary test, one participant used the expression, “It crossed my mind at that moment, this (simulation experience)” [K. A.].

##### 3.3.2.4 Culture

The analysis revealed participant reflections on how socio-cultural factors specific to Türkiye influenced emergency medicine practice as portrayed in the scenarios.

Patient expectations: Participants highlighted the impact of specific societal values (e.g., extramarital pregnancy) on medical practice. One participant noted, “These are somewhat sensitive topics in Turkey… I mean, it’s an issue that involves physician-patient confidentiality, of course” [N. B.].

##### 3.3.2.5 Evaluation of the simulation

Participants provided feedback on the simulation’s realism and the utility of the post-exercise evaluation. Participants evaluated the realism of the simulation and the feedback it provided.

Performance evaluation: Participants reported becoming aware of their deficiencies, such as missing forensic cases and making medication dosage errors. One participant expressed this as, “I administered the wrong adrenaline dose… it was too late, just as it would be in real life” [A. K.].

##### 3.3.2.6 Self-directed learning

The post-simulation debriefing process was described by participants as a trigger for self-directed learning through increased self-awareness.

Awareness: Participants expressed that they became aware of their deficiencies in skills beyond technical knowledge, such as mastering the work environment and team communication. One participant exemplified this awareness by saying, “When I go to a new place, I definitely take a tour of the resuscitation room and make sure where everything is… I decide where to stand by the patient’s head so that I can see the monitor well and also reach the defibrillator” [T. A.].

#### 3.3.3 Participants’ views on the use of simulation in medical education

##### 3.3.3.1 Structure and frequency

Participants unanimously advocated for the systematic integration of simulation training into both undergraduate and postgraduate medical education. Recommendations for frequency suggested that these sessions should be held at least once or twice annually. Regarding the optimal timing within the educational curriculum, participants proposed its implementation during the clinical years of medical school, at the beginning and end of residency training, specifically from the 2.5th year of specialization onwards, and as a recurring element throughout. They emphasized that the simulation content should be tailored to the learner’s context, taking into account their current clinical rotation (e.g., emergency department triage zone), level of seniority, and the specific needs of their training program and institution. This approach would effectively integrate theory with practice, with the number and focus of cases diversified to meet these defined needs.

##### 3.3.3.2 Benefits

Participants described several perceived educational benefits of the simulation methodology. They reported that it encouraged self-directed learning and increased their motivation to seek and receive feedback. Many found the practice useful for identifying and addressing personal knowledge and skill gaps. A commonly noted advantage was the perceived high retention of learning from this experiential approach. The integration of theoretical and practical knowledge within simulations was seen as a powerful tool for improving performance in their current clinical duties and for enhancing their competence in real-world practice after graduation.

##### 3.3.3.3 Content

In addition to the scenarios used in the study, participants suggested several topics for future simulation content. Recommendations focused on managing rare diseases, optimizing patient discharge processes, and improving patient communication. Further suggestions included the management of stable patients, cardiopulmonary resuscitation (with a specific emphasis on resuscitation in pregnancy), bedside ultrasonography, suturing, phlebotomy, and endotracheal intubation. These proposals reflect participant-identified needs for content that spans both complex clinical reasoning and fundamental procedural skills.

## 4 Discussion

This study explored the development and initial implementation of a simulation-based curriculum systematically designed using Kern’s model and investigated its perceived utility for addressing both technical and non-technical skills among emergency medicine residents. Our findings suggest that multi-patient simulations were perceived by participants as effective in replicating high-pressure emergency environments, which aligns with prior studies emphasizing their role in practicing task-switching, prioritization, and crisis management skills (22, 23). Participants reported that the structured debriefing sessions were a critical component, facilitating the transformation of experiential shortcomings into learning opportunities, fostering self-awareness, and providing a forum for recalibrating clinical reasoning (24, 25). The qualitative data indicated that the perceived benefits of this simulation exercise extended beyond procedural proficiency to include empathy, leadership, and resilience in high-stakes situations.

Participants reported stress levels akin to real emergencies, with novices citing simulation inexperience as a compounding factor. While stress increased motivation for pharmacological and procedural review (26), it also prompted defensive practices such as unnecessary imaging in pediatric trauma, reflecting broader malpractice-driven overtesting trends (27). Simulation’s capacity to replicate these pressures offers a safe space to address defensive behaviors, as noted by Guraya et al. (28), who advocate for targeted scenarios to recalibrate clinical decision-making. In parallel, the ectopic pregnancy scenario highlighted participant-identified challenges in maintaining empathy and nonjudgmental communication amid cultural stigma, underscoring the value of culturally immersive simulations (29). Similarly, challenges in managing agitated families during pediatric cases highlight the need for advanced communication training, a gap previously identified in Canadian emergency programs (30).

Leadership and team coordination emerged as critical competencies, with senior residents demonstrating superior crisis resource management. This aligns with previous reports that link effective leadership to reduced medical errors in emergencies (31). Post-simulation debriefings were pivotal in reinforcing these skills, as participants reported improved self-awareness and strategies for workload distribution, consistent with previous findings (32). However, the persistence of stress-induced automaticity in some learners suggests the need for repeated simulations to entrench adaptive behaviors (33).

A key strength of this study is its rigorous, multi-phase development process grounded in Kern’s established curricular framework (13). Participants perceived the use of complex, multi-patient scenarios as reflective of the cognitive demands and contextual challenges encountered in real emergency department settings. In addition, the mixed-methods design supported an in-depth exploration of the learning experience by situating qualitative focus group findings within the broader curriculum development process.

This study has several limitations. The implementation phase involved a small sample of residents from a single institution, with no control group or baseline assessments, limiting generalizability and the ability to attribute perceived improvements solely to the simulation. The reliance on self-reported data from focus groups and debriefings introduces potential social desirability bias and reflects short-term perceptions rather than objective or longitudinal outcomes. Consequently, the long-term effects on clinical behavior, patient outcomes, and skill retention remain unknown. Although the scenarios were designed to approximate real clinical environments, inherent differences between simulation and practice may limit direct transferability, and results may vary with alternative simulation modalities or scenario designs. Notably, the exclusion of certain high-risk scenarios, such as environmental injuries, toxic exposures, and psychiatric emergencies, by local experts contrasts with recommendations that advocate for their inclusion due to their alignment with rare but critical events (34). This discrepancy may reflect localized prioritization of clinical needs or gaps in regional expertise, underscoring the need for context-sensitive curriculum design (35).

For clinical educators, this study provides a validated, practical blueprint for implementing a simulation-based curriculum that addresses critical competencies beyond medical knowledge. Emergency medicine training programs can adopt similar multi-patient, scenario-based exercises to safely expose residents to high-stress decision-making, ethical dilemmas, and communication challenges (35). This approach directly prepares trainees for the realities of clinical practice, potentially reducing errors and enhancing patient safety by building confidence and competence in a risk-free environment.

While participants reported improvements in technical and non-technical skills, its impact on long-term behavioral change remains uncertain. As Tremblay (36) cautions, stress exposure alone does not guarantee knowledge retention or real-world application. Furthermore, the mixed attitudes toward simulation efficacy among some participants, particularly those resistant to altering entrenched practices, echo Schepper et al. (37), who identify institutional culture as a barrier to transformative learning. Future studies should explore longitudinal outcomes, including patient care metrics and malpractice rates, to validate the simulation’s translational benefits.

## 5 Conclusion

This study explores the potential value of simulation-based learning in postgraduate emergency medicine education, consistent with literature demonstrating its benefits for resuscitation skills, crisis resource management, and patient safety. Using expert consensus and the Delphi method, a context-specific simulation program was developed to address emergency training needs in Türkiye. Participants identified multi-patient simulations as particularly effective for preparing for high-stress, multitasking environments and for strengthening non-technical skills such as leadership, teamwork, and communication. Unstructured debriefings were identified as supporting reflective learning and identification of skill gaps, while the integration of ethical dilemmas enhanced perceived competence in managing complex decisions related to confidentiality and informed consent. These findings suggest that well-structured simulations may help cultivate resilient, patient-centered practitioners by addressing competency gaps, ethical challenges, and systemic stressors. However, success hinges on contextual adaptability, robust debriefing frameworks, and alignment with evolving clinical demands. As healthcare complexity grows, simulation must remain dynamic, bridging the chasm between theoretical rigor and the chaotic reality of emergency care.

## Data Availability

The raw data supporting the conclusions of this article will be made available by the authors, without undue reservation.
